# Reductive evolution of virulence repertoire to drive the divergence between community- and hospital-associated methicillin-resistant *Staphylococcus aureus* of the ST1 lineage

**DOI:** 10.1080/21505594.2021.1899616

**Published:** 2021-03-18

**Authors:** Marina Farrel Côrtes, Ana Maria N. Botelho, Paula Terra Bandeira, William Mouton, Cedric Badiou, Michèle Bes, Nicholas C. B. Lima, André Elias R. Soares, Rangel C. Souza, Luiz G. P. Almeida, Patricia Martins-Simoes, Ana T. R. Vasconcelos, Marisa F. Nicolás, Frédéric Laurent, Paul J. Planet, Agnes M. S. Figueiredo

**Affiliations:** a Laboratório de Biologia Molecular de Bactérias, Instituto de Microbiologia Paulo de Góes, Universidade Federal do Rio de Janeiro, Rio de Janeiro, Rio de Janeiro, Brazil; b Faculté de Médecine Lyon Est, Université de Lyon, Domaine de la Buire, Lyon, France; c Centre International de Recherche en Infectiologie (CIRI)―team Pathogénie des Staphylococques―Université Lyon 1, École Normale Supérieure de Lyon, Lyon, France; d Centre National de Référence des Staphylocoques, Institut des Agents Infectieux, Hôpital de la Croix-Rousse, Hospices Civils De Lyon, Lyon, France; e Laboratoire de Bactériologie, Centre de Biologie et de Pathologie Nord, Institut des Agents Infectieux, Hospices Civils de Lyon, Lyon, France; f Laboratório Nacional de Computação Científica, Petrópolis, Rio de Janeiro, Brazil; g Perelman School of Medicine, University of Pennsylvania, Philadelphia, PA, USA; h Children’s Hospital of Philadelphia, Philadelphia, PA, USA; i Sackler Institute of Comparative Genomics, American Museum of Natural History, New York, NY, USA

**Keywords:** Methicillin-resistant *Staphylococcus aureus*, ST1-SCC*mec*IV, comparative genomics, whole genome sequencing, evolution of virulence, evolution of pathogenicity

## Abstract

Methicillin-resistant *Staphylococcus aureus* (MRSA) of the ST1-SCC*mec*IV lineage has been associated with community-acquired (CA) infections in North America and Australia. In Brazil, multi-drug resistant ST1-SCC*mec*IV MRSA has emerged in hospital-associated (HA) diseases in Rio de Janeiro. To understand these epidemiological differences, genomic and phylogenetic analyses were performed. In addition, virulence assays were done for representative CA – and HA-MRSA strains. Despite the conservation of the virulence repertoire, some genes were missing in Brazilian ST1-SCC*mec*IV including *lukSF-PV, fnbB*, and several superantigen-encoded genes. Additionally, CA-MRSA lost the *splDE* while HA-MRSA strains conserved the complete operon. Most of these variable genes were located in mobile genetic elements (MGE). However, conservation and maintenance of MGEs were often observed despite the absence of their associated virulence markers. A Bayesian phylogenetic tree revealed the occurrence of more than one entrance of ST1 strains in Rio de Janeiro. The tree shape and chronology allowed us to infer that the hospital-associated ST1-SCC*mec*IV from Brazil and the community-acquired USA400 from North America are not closely related and that they might have originated from different MSSA strains that independently acquired SCC*mec*IV cassettes. As expected, representatives of ST1 strains from Brazil showed lower cytotoxicity and a greater ability to survive inside human host cells. We suggest that Brazilian ST1-SCC*mec*IV strains have adapted to the hospital setting by reducing virulence and gaining the ability to persist and survive inside host cells. Possibly, these evolutionary strategies may balance the biologic cost of retaining multiple antibiotic resistance genes.

## Background

Community-acquired (CA) infections by methicillin-resistant *Staphylococcus aureus* of the ST1-SCC*mec*IV lineage were initially reported at the end of the 1980s in a remote region in Western Australia. The infections appeared in aboriginal communities in individuals who had no previous contact with hospitals or large urban centers. These Australian ST1-SCC*mec*IV did not produce Panton-Valentine leucocidin (PVL) and were grouped as Western Australia-1 clone (WA-1) [1]. Subsequently, in the USA, in the late 1990s, outbreaks involving PVL-producing isolates, ST1-SCC*mec*IV (named MW2), were reported as causing severe CA infections in children in Minnesota [2]. Years later, new outbreaks of ST1-SCC*mec*IV CA-MRSA (also PVL producers) were reported in the US and Canada; these CA-MRSA strains were named USA400 [3,4]. In Brazil, MRSA strains of ST1-SCC*mec*IV lineage displaying a pulsed field gel electrophoresis (PFGE) pattern very similar or identical to WA-1/MW2/USA400 have emerged in hospitals located in Rio de Janeiro city; however, these ST1 surprisingly did not produce PVL and were related to only hospital-associated infections [5]. Other studies have reported the emergence of multidrug-resistant MRSA belonging to the ST1-SCC*mec*IV lineage in Ireland from 2013–2016; these were all PVL-negative and were mostly involved in hospital-associated infections [6].

Previous studies from our research group compared ST1 MRSA from Brazil (ST1-BR) and the US (ST1-USA) revealing important differences between ST1-BR and ST1-USA. The PVL genes *lukSF-PV* and the enterotoxin genes *sea, sec*, and *sek* were not detected in ST1-BR despite being present in the North American isolates [7]. The hospital-associated ST1-BR was also more resistant to antimicrobial drugs and showed an increased prevalence in nosocomial bloodstream infections while ST1-USA was more susceptible to antimicrobials and infected previously healthy patients from the community, especially associated with skin/soft tissue infections [3,4]. In fact, using bacteremia and foreign body animal models, we demonstrated that ST1-BR seems to be less virulent than ST1-USA [7]. We also showed that ST1-USA has improved fitness in a mouse competitive model when compared with ST1-BR [7].

The main purpose of this study was to inspect ST1 genomes of hospital-associated (HA) MRSA from Brazil and of CA-MRSA from other countries to understand the genomic divergences that may have driven such epidemiological and biological differences. With this in mind, we used comparative whole-genome approaches associated with Bayesian phylogenetic inference to estimate divergence times and uncover genomic events involved in the ST1 evolution. In addition, some virulence properties of ST1 strains were examined using ex vivo and nematode models to investigate the mechanisms that may have led to the divergence between the virulent CA-MRSA and the more attenuated HA-MRSA of the ST1-SCC*mec*IV lineage.

## Methods

### MRSA isolates and strains

A total of 62 MRSA isolates from Rio de Janeiro hospitals were included in this study. These isolates were from different clinical sites including blood (55.0%), bronchial aspirate (6.7%), skin/soft-tissue (3.3%), urinary infection (3.3%), catheter tips (15.0%), and nasal colonization (16.7%). Most isolates (n = 55) were collected from 2005–2009 (Supplementary Tables S1 and S2). The remaining 7 corresponded to more recent isolates (2014–2015) when the incidence of ST1-MRSA in Rio de Janeiro had declined (Supplementary Table S1). We also included representatives of CA-MRSA from the US (strains 2288 and USA400-0051) and Canada (strains 950122 and 111250134) and a *lukSF-PV* positive ST1-MRSA (strain 0515798) nasal isolate from Australia (Supplementary Table S1).

For virulence assays, we selected the archetypal strains of CA-MRSA from the US (strains MW2 and USA400-0051) as well as HA-MRSA from Rio de Janeiro (strains 07–059 and 08–028). These strains were more and less virulent, respectively, in a bacteremia murine model [7]. Our phylogenomic analysis demonstrated that these strains are representative of these two clusters of CA – and HA-MRSA. To analyze a possible relationship between lower cytotoxicity and increased persistence inside osteoblastic cells, we chose the archetypal strain 08–028 (ST1-BR; slightly less virulent than the ST1-BR strain 07–059) and MW2 (ST1-USA; slightly more virulent than the ST1-USA strain USA400-0051). These strains were used in the bacteremia model [7] and the nematode model. Strain MW2 (*lukSF-PV* ^+^) was used as a positive control for the cytotoxicity assays with monocytic cells using the two archetypes of the HA-MRSA from Brazil. Finally, the MW2-cloned *splD* or *splE –* the so-called MW2D and MW2E [8], which carry the *S. aureus* expression vectors pCND (pCN49-P*_blaz_:splD*) or pCNE [pCN49-P*_blaz_:splE*), respectively – were also used for the experiments with osteoblastic cells. The shuttle vector PCN49 was constructed by others [9]. The isogenic strain (MW2B) differs only from clones MW2D and MW2E for the absence of the *splDE* genes [8].

### DNA microarray assay

Total DNA was obtained from Brazilian isolates (n = 55) using a commercial extraction kit (Qiagen, Cat No./ID: 51304; Courtaboeuf, France) according to the manufacturer’s recommendation. The microarray genotyping [*S. aureus* Genotyping Kit 2.0 kit, Alere Technologies; Cat. No. 245200096; Jouy-en-Josas, France) was performed as previously described [10]. The method covers 336 alleles corresponding to 180 genes. MLST assignment was determined as previously described (10]. A hierarchical binary cluster tree was generated by clustering analysis with a distance matrix using Mesquite [11].

### Whole genome sequencing and analysis

In addition to DNA microarrays, whole genome sequencing was carried out for representatives of the Brazilian ST1-MRSA (ST1-BR, 07–059, 08–028, CR 14–005, CR 14–006, CR 14–039, CR 14–040, CHU 15–072, CHU 15–073, and CHU 15–090), the US (ST1-USA, USA400-0051 and 2288), Canada (ST1-CA, 950122 and 111250134), and Australia (ST1-AU, 0515798). Total genomic DNA was obtained using the Wizard Genome DNA Kit (Promega, Cat No. A1120; Madison, WI, USA) following the manufacturer’s instructions. The genome sequences were obtained using Ion Torrent PGM (Life Technologies, Carlsbad, USA) and 454 Platforms Illumina (Illumina, San Diego, USA). In the case of Ion Torrent, the library was constructed using the Ion Xpress Fragment Library Kit with 100 ng of DNA (Life Technologies, Cat No. 4471269, Carlsbad, CA, USA). The Ion Sequencing Kit v2.0 was used following the recommended protocol. The sequencing was carried out on 316 chips using a Torrent Suite 1.5. GS Paired End Adaptor and GS emPCR II Kits (Amplicon A Paired End, Cat No. 05463343001; Roche, Basel, Switzerland) were used to close the gaps. The resulting paired-end libraries were deposited on the Peak Titer Plate for sequencing on the Genome Sequencer FLX System (454 Life Science, Roche). The fragments were processed through a standard protocol for the GS FLX 454 using the GS FLX Sequencing Kit (Roche, Cat No. 5233526001) and the GS PicoTiterPlate as recommended by the manufacturer (Roche, Basel, Switzerland). The sequencing reads were assembled in contigs and scaffolds using the Newbler software (version 2.9, Roche Inc.). The cross_match alignment tool (phred/phrap/consed package) was used to align the contigs to a complete sequence of the genome of *S. aureus* ST1-SCC*mec*IV (MW2) available in GenBank (Acc. BA000033). Gaps within and between the scaffold results from repetitive sequences were solved *in silico*. The annotation of the closed genomes was performed automatically through the SABIA Platform (http://www.sabia.lncc.br/login); in this platform, coding sequences are identified and compared using different nucleotide and protein banks (NCBI-nr, KEGG, InterPro, and UniProtKB.Swiss-Prot).

For Illumina sequencing, libraries were created using the Illumina Nextera DNA XT Kit following the manufacturer’s instructions (Illumina, Cat No. FC-131-1002; San Diego, CA, USA). Libraries were sequenced on an Illumina MiSeq platform (paired end reads of 125 bp). BBduk Trimmer 1.0 was used to trim Nextera adapters, low-quality read-ends with base quality score <35, and discard reads <100 bp. Genome assembly and annotation were performed using PATRIC 3.5.43 (https://www.patricbrc.org/).

A blast ring image generator (BRIG) was used to display circular comparisons between the completely closed genomes and to visualize regions of genetic plasticity (RGPs), gene presence/absence, truncations, and sequence variations [12]. The annotation of prophages was initially performed for the closed genomes using PHAST [13] and by curated annotation using local BLAST and the available phage literature. Virulome and resistome analyses were initially performed with the closed genomes using the VirulenceFinder 1.5 program [14] and ResFinder 3.0 [15], respectively. The associated genes identified were subsequently searched in the draft genomes using local BLAST (http://blast.ncbi.nlm.nih.gov/Blast.cgi). The alignment of genomic regions associated with prophages and genomic islands was visualized using EasyFig [16]. Phylogenetic trees were constructed with 96 ST1 genomes (Table 1 and Supplementary Table S3) based on SNPs using the REALPHY version 1.12 with default parameters. Briefly, the sequences were mapped to the reference sequence (MW2 genome) via Bowtie2. From these initial alignments, multiple sequence alignments were recreated using PhyML for tree construction [17]. The genomes of 4 ST80 strains (SA6–LAU, SA5-LAU, SA12-LAU, and SA7-LAU), whose sequences are available in the GenBank, were also included as outgroups (Supplementary Table S3).

**Table 1.** MRSA strains, BioProject, and GenBank accession numbers of the genomes sequenced in this work

Strain	BioProject	Accession number
0515798	PRJNA577568	CP045474; CP045476
111250134	PRJNA577563	CP045442; CP045443
07–059	PRJNA577559	CP045439; CP045440
08–028	PRJNA577414	CP045435; CP045438
950122	PRJNA577560	CP045441
USA400-0051	PRJNA345484	CP019574.1
2288	PRJNA433086	CP026646.1
CR14-005	PRJNA586869	GC_013423445.1
CR14-006	PRJNA586873	GCA_013423475.1
CR14-039	PRJNA587453	GCA_013423465.1
CR14-040	PRJNA587456	GCA_013423545.1
CHU15-072	PRJNA587457	GCA_013423605.1
CHU15-073	PRJNA668989	JADBPR000000000
CHU15-090	PRJNA587464	GCA_013423575.1

### Bayesian phylogenetic inference

We estimated the phylogeny and divergence time of the alignment of 73 genome sequences using the Bayesian genealogical inference package [BEAST v2.5; 18]. We assumed the HKY+Γ nucleotide substitution model and the constant population model of the coalescent process [19,20]. We calibrated the molecular clock under a relaxed clock [21] and tip dates [19] allowing for different rates on different branches. We combined three independent Markov Chain Monte Carlo (MCMC) runs to ensure proper mixing of the chain. Each chain ran for 200 million iterations, and we discarded the first 10% as burn-in. We visualized convergence of the MCMC chains by eye using Tracer v1.7.1 [22] and calculated the maximum clade credibility tree using TreeAnnotator v2.5 from the BEAST package. The final tree was edited with FigTree v1.4.3 (available at https://github.com/rambaut/figtree).

### In vitro biofilm assays

Biofilm assays were performed in 96-well inert polystyrene microtiter plates (Nunclon; Nunc A/S, Cat No. 150,787; Roskilde, Denmark) using tryptic soy broth (TSB) (BD; Becton, Dickinson and Company, Cat No. 295634; Le Pont de Claix, France) supplemented with 1% (w/v) glucose (TSB-1% Glc) as described previously [23]. Briefly, bacteria were grown in TSB-1% Glc in a shaker (250 rpm) at 37°C for 18 h. Cultures were then diluted 1:100 in TSB-1% Glc (200 μL) and inoculated into each well; plates were incubated at 37°C without shaking for 24 h and 48 h. The biofilm development was assessed by determining the optical densities (OD; 570 nm) of crystal violet-stained biofilms. Biofilm unit (BU) was defined by the formula: BU = OD_B_/OD_G_ where OD_B_ is the OD of the stained biofilm and OD_G_ is the OD of the bacterial cell growth [23].

Biofilm formation was statistically analyzed by two-way ANOVA. Since the ST1-BR strains carry the genes for SplDE proteases, to evaluate the occurrence of biofilm disintegration/detachment after 48 h of biofilm growth, the 24 h-biofilm of each ST1 strain analyzed was considered the control (initial biofilm) and the correspondent 48 h-biofilm the test (final biofilm). To analyze the significance of the differences in the biofilm accumulated between control (24 h) versus test [48 h), Sidak’s test for multiple comparisons was applied following two-way ANOVA. The post-hoc Tukey’s test was used to evaluate significant interstrain differences in biofilms developed after 24 h and 48 h of biofilm growth. These tests were performed for significance level of α = 0.05 using GraphPad Prisma v.8.4.3.

### Interactions of ST1-MRSA with human osteoblasts

These assays were performed as recommended previously [24] using the immortalized human MG-63 osteoblast-like cells (ATCC CRL-1427; GIBCO, Paisley, UK] cultured in complete culture medium (CCM) consisting of Dulbecco’s modified Eagle’s medium (DMEM, GIBCO, Cat No. 31053028; Paisley, UK) containing 2 mM L-glutamine and 25 mM HEPES supplemented with 10% (v/v) fetal bovine serum (SFB; GIBCO, Cat No. 16000036), 100 U/mL penicillin, and 100 mg/L streptomycin. Briefly, monolayers of osteoblasts were washed with GIBCO PBS phosphate-buffered saline (GIBCO, Cat No. 10010023). The bacterial strains were grown at 37°C to the mid-exponential phase in brain heart infusion broth (BHI, bioMérieux, Cat No. 51009). After centrifugation, bacterial pellets were suspended in supplemented DMEM without antibiotics and were added to the monolayers at a multiplicity of infection (MOI) of 0.01. For adhesion assays, the infected osteoblasts were collected after a 2 h-incubation, washed twice with PBS, and lysed with sterile water (10 min at room temperature). After repetitive pipetting, colony-forming units (CFU) were determined by plating serial dilutions on BHI agar. Internalized bacteria were assessed after 3 h of incubation. Adhered bacteria were eliminated by the treatment of infected osteoblasts with 200 μg/mL gentamicin for 1 h. After washing with PBS, the osteoblasts were lysed and plated on for CFU determinations. For intracellular persistence and cytotoxicity assays, the adhered bacterial cells were removed, and infected cells were incubated for 24 h and 48 h in supplemented media containing 40 μg/mL gentamicin as the only antibiotic. After incubation, cell cytotoxicity was measured in the supernatant of infected cultures by determining lactate dehydrogenase (LDH) using a colorimetric method and an automated clinical chemistry analyzer (Dimension Vista System; Siemens Healthcare Diagnostics, Tarrytown, NY). In experiments with the *spl* clones, the LDH was measured using a cytotoxicity detection kit LDH (Roche, Cat No. 11644793001) according to the manufacturer’s recommendations. In parallel, the infected osteoblasts were lysed for CFU determinations. The percentage of adhered and internalized bacteria were calculated by setting the bacterial inoculum size to 100%. The rate of bacteria that persisted within osteoblastic cells was calculated by setting the number of internalized bacteria to 100%. For cytotoxicity assays, the percentages were calculated on the basis of the LDH measurement after treatment of the osteoblastic cells with 1% (v/v) triton X-100 (Merck KgaA, Cat No. 9002–93-1; Darmstadt, Germany), which was defined as 100% lysis. Statistical analysis of the adherence and internalization data were performed using unpaired two-tailed Student t test with GraphPad Prisma v.8.4.3.

Bayesian inference was also applied as an alternative to the null hypothesis by calculating Bayes factor with Jeffrey’s Zellner-Siow Cauchy prior (JZS-BF) for r = 0.707 and the Bayes Factor Package R v.3.3.2. Two-way ANOVA followed by Turkey’s test was applied using GraphPad Prisma v.8.4.3 for persistence and cytotoxicity assays (24 h and 48 h) with the strains 08–028 and MW2. Turkey’s test following one-way ANOVA was performed for the experiments with *splDE* mutants. The significance was tested at α = 0.05 for all experiments.

### Cytotoxicity assessment in monocyte cells

The human monocyte U937-C5R1a cell line [25] was cultured in RPMI (GIBCO, Cat No. 11875) supplemented with 10% (v/v) BFS. Cytotoxicity was analyzed as previously described [26]. Briefly, monocytes were standardized to contain 1.25 × 10^6^ cells/mL, and then 2.5% propidium iodide (PI) was added. The strains were cultured in 5 mL CCY medium (3% yeast extract, 2% casamino acids, 2.3% pyruvic acid, 0.63% Na_2_HPO_4_, and 0.041% KH_2_PO_4_, pH 6.7). Monocyte death was measured every 10 min for 6 h by determining PI uptake using TECAN (Männedorf; Switzerland). Two-way repeated measure ANOVA was performed at a significance level of 0.05 using GraphPad Prisma v.8.4.3.

### Nematode survival assays

Wild-type *C. elegans* Bristol strain N2 was obtained from the *Caenorhabditis* Genetics Center and was grown on nematode growth medium [NGM) seeded with *Escherichia coli* strain OP50 at 25°C as described previously [27]. The ST1-BR strains 07–059 and 08–028 and the ST1-USA strains MW2 and USA400-0051 were grown overnight on 3.5-cm-diameter plates containing tryptic soy agar (TSA; BD, Cat No. 221239, Le Pont de Claix, France] supplemented with 5 μg/ml nalidixic acid (TSA-NA). Plates were incubated for 4 h at 37°C and cooled to room temperature. The Bristol N2 wild-type *C. elegans* strain was grown on NGM in plates containing bacterial lawn (*E. coli* OP50) as the food source.

The nematodes were collected by washing the plates with M9 buffer supplemented with nalidixic acid at 5 μg/ml. Then 30 to 40 nematodes in the L4 stage were transferred to TSA-NA plates containing the bacterial growth to be assayed [28]. The plates were incubated at 25°C and monitored every 24 h for live and dead worms for four days. The negative control was the nonpathogenic *E. coli* strain 0P50. The nematode survival curves were analyzed using Kaplan-Meier method and compared statistically with the log-rank (Mantel-Cox) test using GraphPad Prisma 8.4.3. A *p*-value < 0.05 was considered statistically significant.

## Results

### Virulence genes

**Figure 1.**
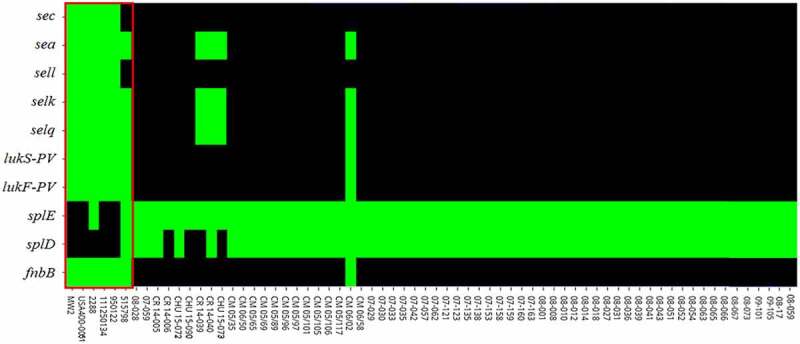
Heatmap of virulence genes heterogeneously distributed amongst ST1 strains obtained by DNA microarrays and BLAST searches. **Green**: gene presence and **black**: gene absence. ***fnbB*** encoding fibronectin-binding protein B; ***spl****D* and ***spl****E* encoding serine proteases D and E; ***lukF-PV*** and ***lukS-PV*** encoding Panton-Valentine leucocidin subunits F and S; ***selq, sel****k* and ***sel****l* encoding staphylococcal enterotoxin-like Q, K and L; ***se****a* and ***se***c encoding staphylococcal enterotoxin A and C. Virulence factors for the strains MW2, USA400-0051 and 2288 (EUA), 111250134 and 950122 (Canada), 0515798 (Australia) and for the Brazilian strains with the prefix CR or CHU were only assessed by BLAST search. For the remaining 55 strains (BR), the virulence factors were assessed using both genomic tools. Red rectangle comprises the ST1-INT. All other are ST1-BR

DNA microarray analysis showed that not only were *lukSF-PV* genes absent in the genome of ST1-BR (HA-MRSA) but also the genes encoding fibronectin-binding protein B (*fnbB*), enterotoxins (*sea* and *sec*), and enterotoxin-like superantigens (*selk, sell* and *selq*) were lost. Most ST1-INT analyzed carried these genes (Figure 1). Conversely, only ST1-BR (with exception of CR 14–006, CHU 15–090, CR 14–039, and CHU 15–073) and ST1-AU harbored the complete *splABCDEF* operon. Amongst all ST1-BR tested by DNA microarrays, only one (CM 06/02) had *lukSF-PV* genes. However, the virulence signature of this MRSA was identical to that of ST1-AU 0515798 potentially indicating international spread (Figure 1).

To further confirm these data and to understand the genetic context of the genomic regions that normally carry these genes, whole genome sequencing was performed for representatives of ST1-SCC*mec*IV. The BioProject and GenBank accession numbers are presented in Table 1. The sizes of the closed, completed ST1 genomes (ST1c) varied from 2,809,177 bp (ST1c-CA; strain 950122) to 2,936,025 bp (ST1c-BR; strain 08–028) with %GC content varying from 32.79 to 32.88 (Table 2). The Brazilian strains and the ST1c-CA strain 111250134 have the largest genomes.

**Table 2.** Characteristics of the completely closed genomes of the MRSA strains belonging to ST1-SCC*mec* IV

Strain	Genome Size (bp)	%GC	Plasmid	%Identity^a^	SNP^a^	Reference
**MW2**	2,820,462	32.82	1	100	0	[29]
**USA400-0051**	2,832,530	32.79	1	99.7	45	This study
**2288**	2,814,563	32.79	0	99.5	650	This study
**08–028**	2,936,025	32.87	3	98.1	2009	This study
**07–059**	2,910,390	32.88	1	98.2	1884	This study
**950122**	2,809,177	32.79	0	99.6	267	This study
**111250134**	2,932,476	32.85	1	99.7	368	This study
**0515798**	2,838,852	32.80	2	98.8	1332	This study

^a^SNP, single nucleotide polymorphism. Those values were calculated using MW2 as reference genome.

**Figure 2.**
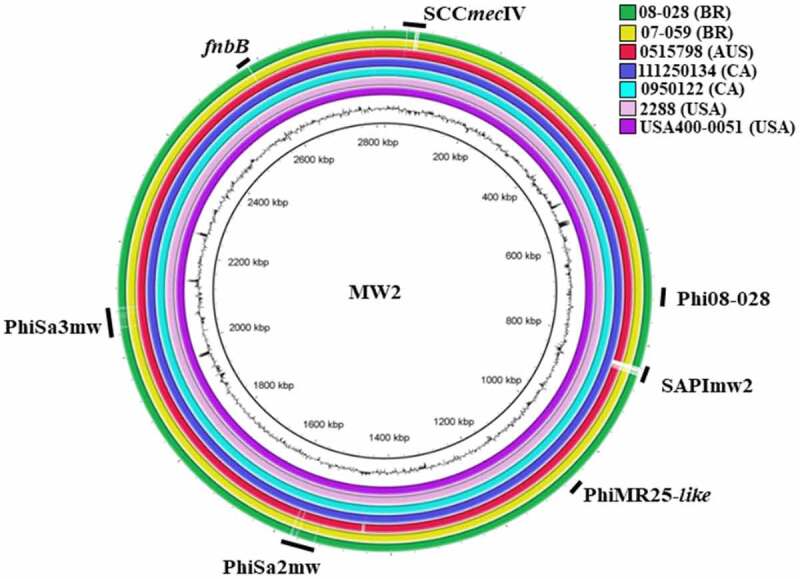
Overview of the completely closed genomes of the analyzed ST1-SCC*mec*IV MRSA with the location of prophages, genomic islands and the *fnbB* gene. The color circles represent the genomes of each sequenced strain. The outer black circle represents the GC content, and the inner circle is the reference genome of the archetypal CA-MRSA strain MW2. **SCC*mec***: all strains carry SCC*mec* IV. **Phi08-028**: bacteriophage only present in the genome of the strain 08–028. **SAPImw2**: *S. aureus* pathogenicity island, mw2, present in all strains except 08–028, 07–059, and 0515798. **PhiMR25-*like***: bacteriophage present in all strains except 08–028, 07–059, and 111250134. **PhiSa2mw** and **PhiSa3mw**: bacteriophage present in the genomes of all strains analyzed. ***fnbB* gene**: present in all ST1-SCC*mec*IV genomes analyzed except in the genomes of the strains 08–028, and 07–059 (HA-MRSA)

Using the genome of strain MW2 (ST1c-USA) as a reference, the sequenced genomes showed high nucleotide identity varying from 98.1% for ST1c-BR, strain 08–028 to 99.7% for ST1c-USA, strain USA400-0051 (Table 2). The analysis of the completely closed genomes using BRIG revealed that they shared most genetic loci. However, some differences in the regions of genomic plasticity (RGPs) that corresponded to phages, the pathogenicity island SAPImw2 and SCC*mec* were mapped (Figure 2). The core genome was defined as the set of genes common to all genomes and contained 2,280 genes. In total, 50 genes were exclusively present in ST1c-BR. Of those, there were 20 encoded proteins with an unknown function (hypothetical proteins) and 30 encoded proteins related to mobile genetic elements (MGE) including resistance genes. The ST1c-BR and ST1c-AU strain 0515798 were *spa* type t127 while the ST1c-USA genomes were *spa* type t128 and t125 (Supplementary Table S1).

**Figure 3.**
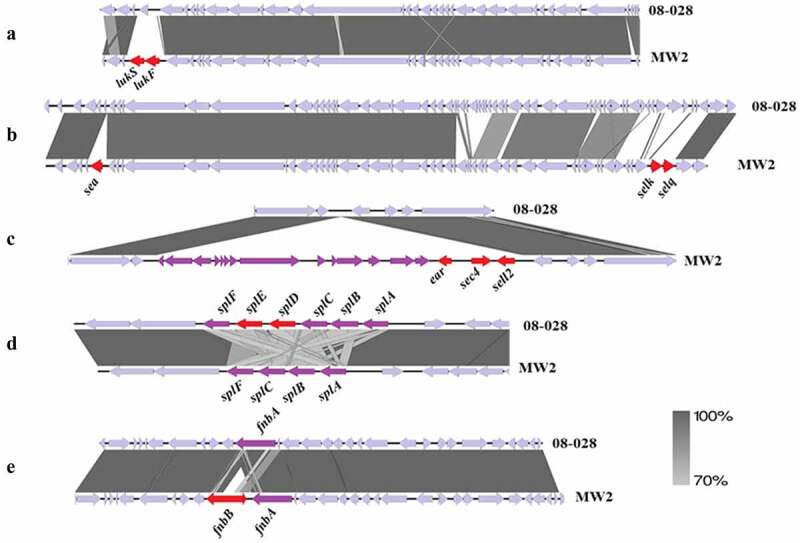
Comparison of the genetic context of the regions of genomic plasticity between ST1-BR (HA-MRSA) and ST1-USA (CA-MRSA). The figure shows the alignment of the representative strains from Brazil (08–028) and from the USA (MW2). The important missing genes are highlighted in red. Note that the DNA identity between these regions is higher than 70%. **A**. Phage-related MGE (PhiSa2mw) region carrying *lukSF-PV* genes (encoding PVL), which are missing in the ST1-BR. **B**. Phage-related MGE (PhiSa3mw) carrying *sea, selk*, and *selq* genes, which are missing in the ST1-BR. **C**. Pathogenicity island (SAPImw2) carrying *ear, sec*, and *sell* genes in MW2 (ST1-USA). Note that this island is absent in the ST1-BR. **D**. Genomic island νSaβ carrying the operon *splABCDEF* that is only complete in the ST1-BR. **E**. Genomic region encompassing the gene *fnbB*, which is missing in ST1-BR genomes

Genomic analysis corroborated the microarray results that the virulence genes *sea, sec, sell, selq, selk, lukSF-PV*, and *fnbB* were missing in the ST1c-BR genomes and showed that another gene encoding *Escherichia coli* ampicillin resistance protein homolog (*ear*) was present in ST1c-INT but absent in ST1c-BR. In addition, STc-BR had a complete *spl* operon, which was absent in ST1c-INT. These variable genes were mostly located in RGPs such as putative bacteriophages or genomic islands. Surprisingly, these genomic elements presented high nucleotide identity (varying from 98.5% identity and 86% coverage for PhiSa3MW to 99.67% identity and 97% coverage for νSaβ) regardless of the presence or absence of the virulence-associated gene identified as a variable in the ST1 strains (Figure 3). In the closed genomes of international ST1 (ST1c-INT), the *lukFS-PV* locus is located in the phage PhiSa2mw (Figure 3a); *sea, selk*, and *selq* genes are inserted in PhiSa3mw (Figure 3b) while *ear, sec*, and *sell* are located in the SAPImw2 island (Figure 3c); the *splD* and *splE* genes encoding Spl serine proteases were in the genomic island νSaβ (Figure 3d). The genomic region of the *fnbB* gene in the bacterial genome is represented in Figure 3e.

Once the genetic differences between the ST1c-BR and ST1c-INT genomes were recognized, we investigated these differences using local BLAST in other ST1-RJ assembled genomes as well as in 81 genomes of ST1-INT deposited in the GenBank (Supplementary Table S3). This search revealed that ST1-BR showed a unique virulence signature (BR signature) represented by (*lukSF-PV ^–^, ear ^–^, sea ^–^, sec ^–^, sell ^–^, selq ^–^, selk ^–^*) and complete *spl* operon (*spl*_c_). Exceptions were observed for CR 14–040, CHU 15–073, and CR 14–039, which, unlike most ST1-BR, harbor a PhiSa3mw carrying *sea, selk*, and *selq* similar to ST1c-AU strain 0515798. However, unlike ST1c-AU, these ST1-BR do not carry *lukSF-PV* genes. In addition, ST1-BR CR 14–006, CHU 15–090, CR 14–039, and CHU 15–073 lack the *splD* gene.

### Virulence signature and lineage diversification

**Figure 4.**
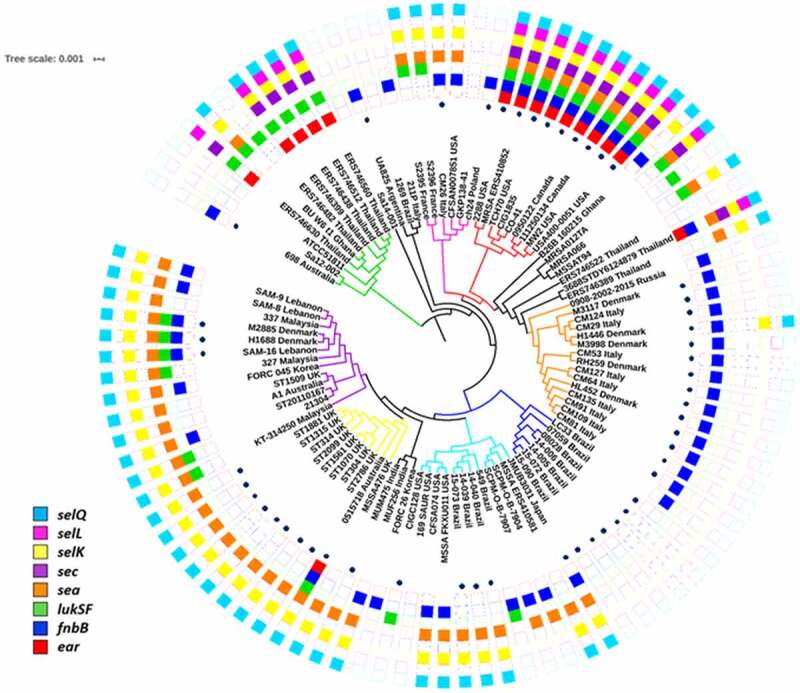
Maximum likelihood phylogenetic tree of ST1-SCC*mec*IV lineage of MRSA. The tree was rooted using the genomes of the ST80 strains SA5-LAU, SA7-LAU, SA12-LAU and SA6-LAU (not shown). The main subclades were highlighted with colors. The heatmap represents the presence (full boxes) and absence (empty boxes) of virulence genes. Dots represent the presence of *mecA* gene

The maximum likelihood (ML) tree topology showed the dark blue subclade grouped strains from Rio de Janeiro with a gene signature (*lukSF-PV ^–^, ear ^–^, fnbB* ^–^, *sea ^–^, sec ^–^, sell ^–^. selq ^–^*, *selk* ^–^). This subclade also clustered the strain LC33 isolated from human milk in the state of Bahia (Figure 4). A second entrance of ST1-BR was grouped in the light blue subclade (CR14-039, CR 15–040, CHU 15–073) together with strains from the US, Thailand (TH), Japan, and another ST1 from Brazil (strain 549) isolated from human blood in Paraná state. The genomes found in the red subclade mostly originated in the US and Canada and carried all the genes recorded for the virulence gene signature (*lukSF-PV ^+^, ear ^+^, fnbB* ^+^, *sea ^+^, sec ^+^, sell^+^, selq^+^, selK^+^*) but lack *splDE* genes (Figures 1 and Figures 4). These strains are closely related to the well-characterized CA-MRSA archetypal clone MW2/USA400 (strains MW2 and USA400-0051) that preserved *lukSF-PV* genes and other important virulence genes. The AU strain 0515798 clustered in the yellow subclade together with strains from the UK (Figure 4). The independent and more basal (green) clade was largely composed of TH strains carrying *lukSF-PV* genes (Figure 4).

It seems more likely that the Brazilians ST1 have conserved the complete operon while the Americans CA-MRSA have lost *splDE* genes based on the following observations: (i) *splDE* genes are part of a gene cluster (*splABCDEF*), (ii) in the tips of the main three branches of the phylogenetic trees there are descendants that maintained the complete *spl* operon [e.g.: ERS746389_Thailanda and ERS746522_Thailanda (black clade), GKP138_41 (pink clade), ST314 and ST1881 (yellow clade)], and (iii) some genomes grouped in the more basal (green) clade equally carried the complete *spl* operon (e.g.: SA12002_Australia, ERS746399_Thailand, BU_WU_t1_Ghana, and ERS746630_Thailand).

The phylogenetic tree obtained under the Bayesian framework was reconstructed using 73 ST1 genomes from different geographic regions including those sequenced in this study as well as genome sequences deposited in the GenBank for which the isolation date was reported. Contrary to our initial hypothesis, the phylogenetic inferences suggest that ST1-BR and ST1-USA are not closely related and probably have a shared common ancestor at about 140 years ago (95% HPD, 1976–1853). The expansion of ST1 CA-MRSA in the US and Canada was estimated to have occurred in 1990 (95% HDP, 1996–1980). The dark blue subclade of the BR strains emerged around 2001 (95% HPD, 2007–1990; Supplementary Figure S1). This subclade grouped the strains displaying the typical BR signature (CR 14–005, CHU 15–090, CHU 15–072, CR 14–006, and the archetypes 07–059, and 08–028) and represents the majority of ST1-BR (93.5%) in the entire collection of 62 ST1 from Rio de Janeiro as revealed by the hierarchical binary cluster tree with DNA microarray data (Supplementary Figure S2) as well as the phylogenetic trees based on SNP calling (Figure 4 and Supplementary Figure S1).

The Bayesian tree topology also confirmed the occurrence of at least two introductions of ST1 MRSA in Rio de Janeiro. Indeed, it was estimated that the BR subclades dark and light blue diverged from around 90 years ago (95% HPD, 1990–1887). The divergence of ST1-BR (blue subclades) from ST1-UK (yellow subclade) was estimated to have occurred approximately 100 years ago (95% HPD, 1984–1882).

### Antimicrobial resistance traits

Analysis of the ST1 genomes showed that the SCC*mec*IV sequences presented three different structures (Supplementary Figure S3). The SCC*mec* of ST1c-BR is 28,749 bp and had an inserted plasmid (pUB110) flanked by IS*431* downstream of the *mecA* gene. This plasmid carries the genes *ble* (resistance to bleomycin) and *aadD* (resistance to aminoglycosides). However, isolate 2288 (ST1c-USA) has the plasmid pT181 carrying the *tetK* gene for tetracycline and doxycycline resistance inserted into its SCC*mec*IV. In addition, ST1c-AU 0515798 contains an insertion of the non-*mec* chromosomal cassette SCC476, which carries a fusidic acid resistance gene, *fusC*.

The antimicrobial resistance genes in ST1-BR were screened using DNA microarray analysis performed for 55 of the ST1-BR isolates (Supplementary Table S2). In addition to *mecA* and *blaZ*, the *aadD* gene encoding aminoglycoside adenyltransferase was highly prevalent (90.7%) in ST1-BR genomes followed by *ermC* encoding rRNA adenine methyltransferase (88.5%), *aphA3* encoding an aminoglycoside phosphotransferase (77.8%), *sat* encoding streptothricine acetyltransferase (77.8%), and *cat* encoding chloramphenicol acetyltransferase (66.7%). The *aacA-aphD* gene, encoding a bifunctional enzyme Aac/Aph associated with aminoglycoside resistance, was detected at a lower frequency (24.1%), as were the tetracycline resistance gene *tetK* (1.9%) and the Q6GD50 putative gene for fusidic acid resistance (1.9%) (Supplementary Figure S4). ST1c-BR also showed intrinsic ciprofloxacin resistance due to substitutions in *grlA* [TCC (Ser-80) → TAC (Tyr)] and *gyrA* [TCA (Ser-84) → TTA (Leu)] genes encoding DNA topoisomerase and DNA gyrase, respectively. Additionally, ST1c-BR and ST1c-INT genomes were screened for resistance traits. Some resistance genes in ST1c-BR genomes such as the genes conferring resistance to bacitracin (*bacA*), streptomycin (*ant(6)-Ia*), aminoglycosides (*aphA*), and arsenic (*acr3*) were located in a plasmid similar to pMW2 in ST1c-BR (07–059 and 08–028). Also, the *qacA* gene encoding an efflux pump that confers chlorhexidine resistance in strain 111250134 (ST1c-CA) was in a pMW2-like plasmid.

### Biofilm accumulation

**Figure 5.**
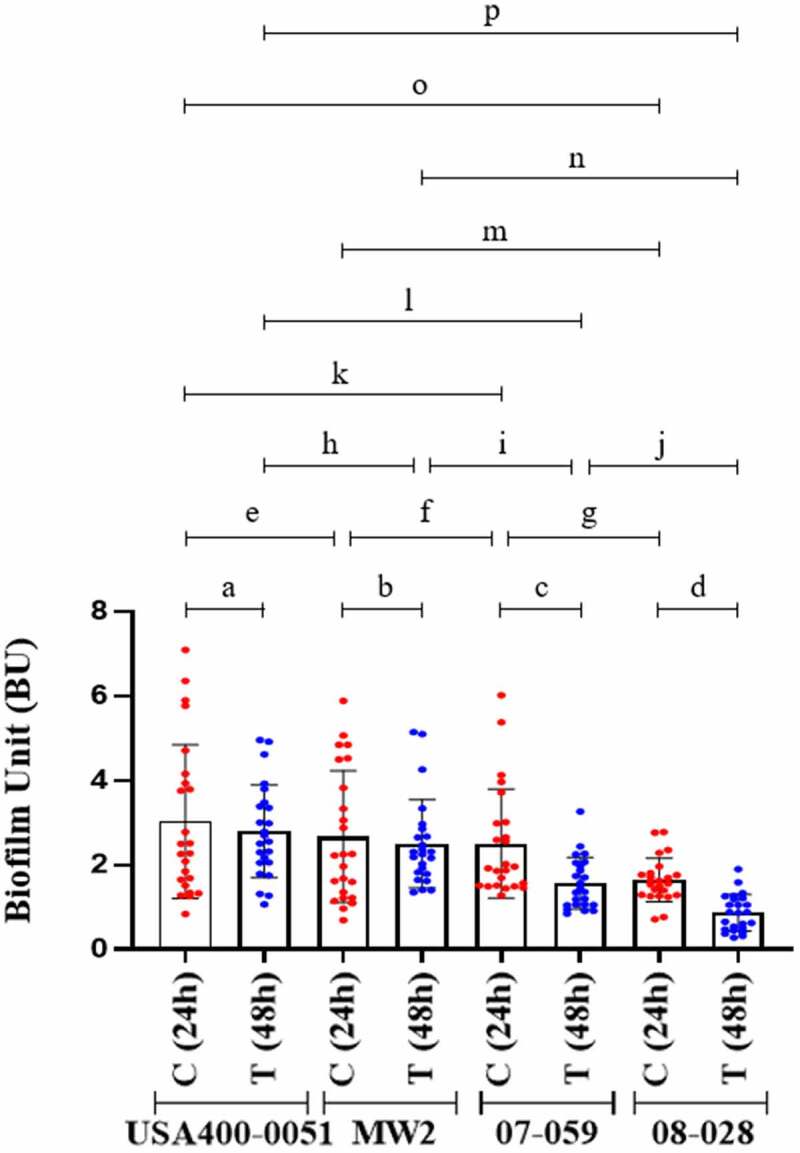
Biofilm accumulation after 24 h and 48 h of biofilm growth. Strain USA400-0051 and MW2 are CA-MRSA (ST1-USA) and strains 07–059 and 08–028 are HA-MRSA (ST1-BR). For each MRSA strain tested, three experimental units were set with eight biological replicates in each. One OD measurement was determined for each biofilm formed (n = 24). Two-way ANOVA was applied for statistical calculation (row factor_(time)_ *p *= 0.0060, df = 1, f = 9.153; column factor_(strains)_ *p *< 0.0001, df = 3, f = 20.50). To evaluate biofilm disintegration/detachment, a reduction in biofilm accumulation was analyzed by Sidak’s multiple comparisons following ANOVA to compare each test (T; 48 h-biofilm) versus the respective control (C; 24 h-biofilm). **a**. *p *= 0.9783, **b**. *p *= 0.9911, **c**. *p *= 0.0011, **d**. *p *< 0.0001. Tukey’s test for multiple comparisons was also applied following ANOVA to evaluate interstrain differences in biofilms formed after 24 h and 48 h of biofilm growth. **e**. *p *= 0.8271, **f**. *p *= 0.9733, **g**. *p *= 0.0239, **h**. *p *= 0.8395, **i**. *p *= 0.0145, **j**. *p *= 0.0004, **k**. *p *= 0.7339, **l**. *p *= 0.0001, **m**. *p *= 0.0168, **n**. *p*.<0.0001, **o**. *p *= 0.0039, **p**. *p*.< 0.0001. The error bar represents the standard deviation

A decrease in biofilm accumulation by the ST1-BR archetypes (Figure 5) was detected when 48 h-biofilm was compared with 24 h-biofilm (08–028: BU_24h_ = 1.638 ± 0.5270 and BU_48h_ = 0.850 ± 0.461, and 07–059: BU_24h_ = 2.501 ± 1.291 and BU_48h_ = 1.564 ± 0.611). However, no reduction in biofilm accumulation was observed for the ST1-USA archetypes, which lack *splDE* genes (Figure 5). The accumulated biofilms for the US strains remained mostly intact with no important differences after 48 h (MW2: BU_24h_ = 2.665 ± 1.562 and BU_48h_ = 2.498 ± 1.043, and USA400-0051: BU_24h_ = 3.023 ± 1.821 and BU_48h_ = 2.791 ± 1.098). When the biofilms formed by ST1-USA versus ST1-BR were compared, interstrain differences were accentuated for the biofilms accumulated at 48 h, compared with those accumulated at 24 h; this result emphases the occurrence of some degradation/detachment of biofilms formed by the Brazilian strains (*splDE* positives) (Figure 5).

### Interactions with osteoblastic cells

**Figure 6.**
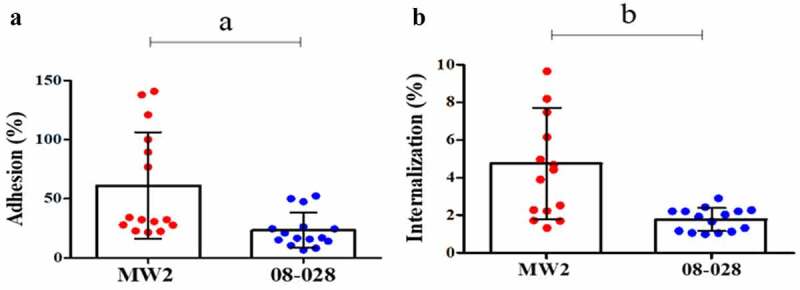
Adherence (a) and invasion (b) assays with the strains 08–028 (ST1-BR; HA-MRSA) and MW2 (ST1-USA; CA-MRSA) using the human osteoblastic cells, MG26. Unpaired two-tailed t test and Bayes factor calculation (JZS-BF) were applied (**a**. *p *= 0.0045, t = 3.085, F = 9.08, df = 28, JZS-BF = 3.9768; **b**. *p *= 0.0007, t = 3.822, F = 26.36, df = 28, JZS-BF = 8.3241). In both cases the calculated Bayes factor was in favor of the alternative hypothesis. For both assays five experimental units were set with three biological replicates in each. Two CFU assays were performed for each biological replicate. Each data point is the mean of two CFU determinations (n = 15). The Error bar represents the standard deviation

**Figure 7.**
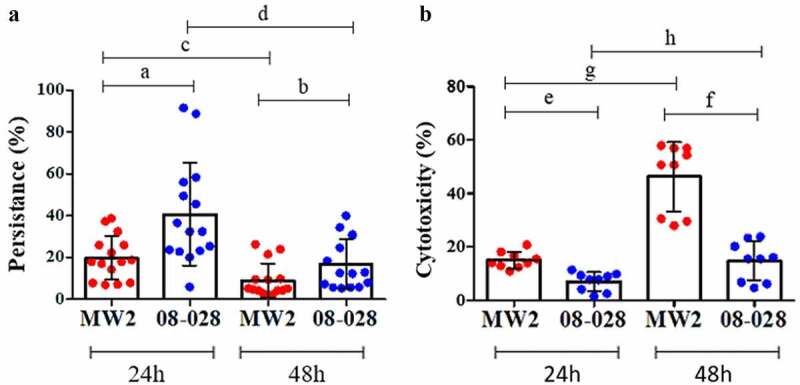
Intracellular persistence and cytotoxicity of MW2 (ST1-USA; CA-MRSA) and 08–028 (ST1-BR; HA-MRSA) for human osteoblastic cells, MG26, determined at 24 h and 48 h. **A**. Persistence data was analyzed by two-way ANOVA (row factor_(time)_ *p *= <0.0001, f = 48.93, df = 1; column factor_(strain)_ *p *< 0.0001, f = 33.65, df = 1) followed by Tukey´s test (**a**. *p *< 0.0001, **b**. *p *= 0.0710, **c**. *p *= 0.0110; **d**. *p <* 0.0001). Five experimental units were set with three biological replicates in each. For each biological replicate two CFU assays were performed. Each data point represents the mean of two CFU determinations (n = 15). **B**. Cytotoxicity assays were tested by two-way ANOVA (row factor_(time)_ *p *= 0.0001, f = 45.48, df = 1; column factor_(strain)_ *p* < 0.0001, F = 63.13, df = 1) followed by Tukey´s test (**e**. *p *= 0.0328, **f**. *p *< 0.0001, **g**. *p *= < 0.0001, **h**. *p *< 0.047). Three experimental units were set with three biological replicates in each. Only one measurement was taken for each biological replicate (n = 9). The error bar represents the standard deviation

The archetypal strain 08–028 (ST1-BR) showed lower adhesion rate (21.6%) when compared with strain MW2 (ST1-USA; 56.4%). There was also a diminished potential for being internalized (1.8% and 4.8%, respectively) by osteoblast cells (Figure 6a and Figure 6b). It is notable that 08–028 showed a higher ability to persist intracellularly after both 24 h (40.6% versus 19.7%) and 48 h (16.8% versus 8.8%) when compared with MW2 (Figure 7a). Cytotoxicity was higher in the cell culture supernatants infected with the strain MW2 when compared with 08–028, at both 24 h (15.05% versus 7.03%) and 48 h (46.3% versus 20.5%) from the onset of the cell culture infections (Figure 7b).

The persistence rate decreased at 48 h for both strains (MW2 and 08–028). In contrast, cytotoxicity increased at 48 h for both (Figure 7a). The ST1-BR 08–028 strain showed higher ability to persist when compared with MW2, mainly at 24 h when the persistence rates were higher. On the other hand, the ST1-USA strain was comparatively more toxic at 48 h when it achieved its highest toxicity (Figure 7b).

**Figure 8.**
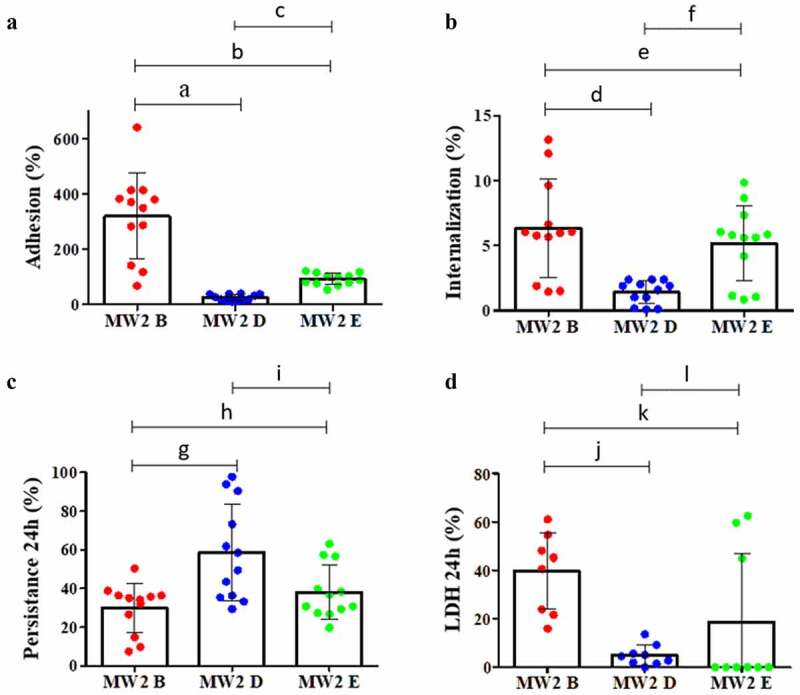
Interactions of MW2-cloned *splD* or *splE* with human osteoblastic cells, MG26. **A**. Adhesion. One-way ANOVA (*p *< 0.0001, F = 34.30, df = 2) was applied followed by Tukey’s test (**a**. *p *< 0.0001, **b**. *p *< 0.0001, **c**. *p *= 0.1856). **B**. Internalization. One-way ANOVA (*p *< 0.0004, F = 10.01, df = 2) was applied followed by Tukey’s test (**d**. *p *= 0.0004, **e**. *p *= 0.5768, **f**. *p *= 0.0069). **C**. Intracellular persistence. One-way ANOVA (*p *< 0.0025, F = 7.203, df = 2) was applied followed by Tukey’s test (**g**. *p *= 0.0035, **h**. *p *= 0.8423, **i**. *p *= 0.0148). **D**. Cytotoxicity measured by lactate dehydrogenase (LDH) assay. One-way ANOVA (p < 0.0016, F = 8.485, df = 2) was applied followed by Tukey’s test (**j**. *p ***= **0.0013, **k**. *p *= 0.0381, **l**. p = 0.3384). **A, B** and **C**. Four experimental units were set with three biological replicates in each. Two CFU assays were performed for each biological replicate. Each data point is the mean of two CFU determinations (n = 12). **D**. Three experimental units were set with three biological replicates in each. Only one measurement was taken for each biological replicate (n = 9). **MW2D** (pCN49-P*_blaz_:splD*); **MW2E** (pCN49-P*_blaz_:splE*); **MW2B** (isogenic strain). The error bar represents the standard deviation

Notably, as observed for ST1-BR archetypes, the strain MW2D also had an important decrease in adherence and invasion (Figure 8a and Figure 8b), and a considerable reduction (>6 times, in the amount of LDH with parallel increase in *S. aureus* intercellular persistence (Figure 8c and Figure 8d). A similar phenomenon was also observed for MW2E – mainly for adherence, but this was not as intense as those detected for MW2D (Figure 8a–Figure 8d).

### Cytotoxicity assay using monoblastic cells

**Figure 9.**
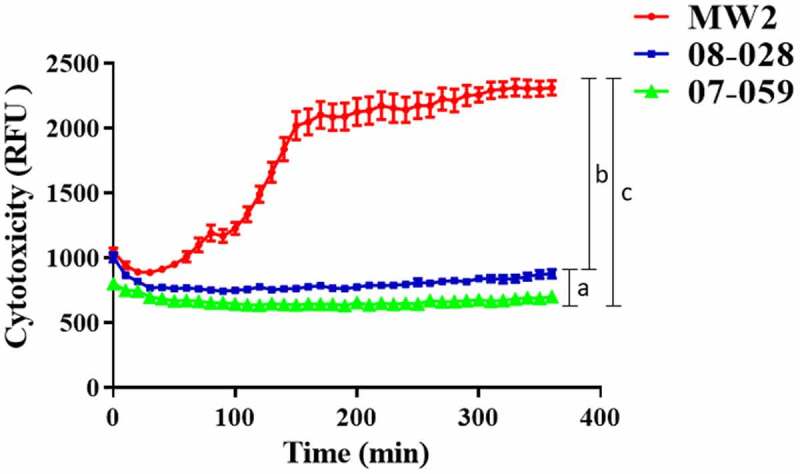
Cytotoxicity of the strains 08–028 and 07–059 (HA-MRSA; ST1-BR) for U937-C5aR monocytic cells, determined by propidium iodate incorporation. The US strain MW2 (*lukSF-PV* ^+^; CA-MRSA) was used as a positive control. Each time point represents the mean of three biological replicates, and only one measurement was taken for each biological replicate (n = 37). For statistical analysis, a two-way repeated measure ANOVA was applied (raw factor_(time)_ *p *< 0.0001, f = 136.7, df = 36; column factor_(strain)_ *p *< 0.0001, f = 258.0, df = 2). Tukey’s test following ANOVA was applied for multiple comparisons at 360 min (**a**. *p *< 0.0001, **b**. *p *< 0.0001, **c**. *p *< 0.0038). RFU: relative fluorescence unit. The error bar represents standard deviation

Supernatants from the Brazilian strains 07–059 and 08–028 (ST1-BR) showed virtually no cytotoxicity activity for the U937-C5aR monoblastic cell line, even at 360 min. However, the MW2 strain was highly cytotoxic for this cell line and achieved a maximum cytotoxicity at 150 min from the onset of the assay. The cytotoxicity remained through the end of the experiment at 360 min (Figure 9).

### C. elegans *survival model*

**Figure 10.**
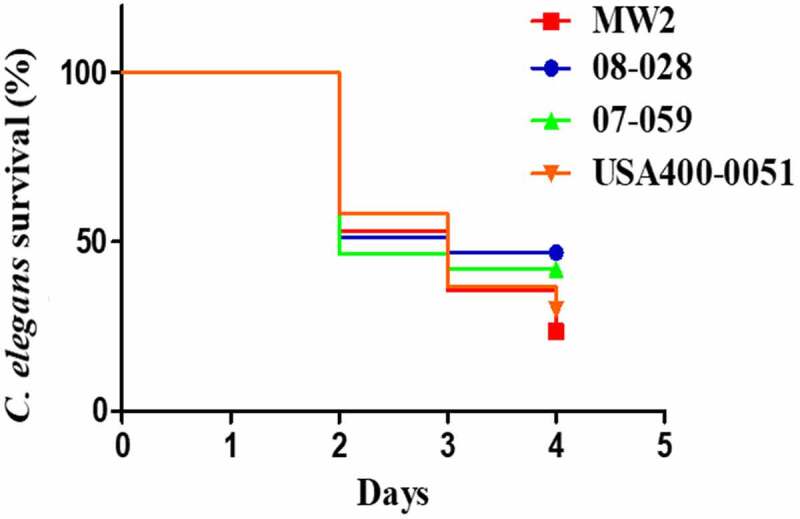
Survival curves for nematode *C. elegans* fed with the strains MW2 and USA400-0051 (CA-MRSA, ST1-USA), and 08–028 and 07–059 (HA-MRSA, ST1-BR). The curves represent animal survival when fed with each one of these strains individually. The Kaplan-Meier method was applied to construct the survival curves, and the log-rank (Mantel-Cox) test used for curve comparisons (*p *= 0.0002; df = 3, Chi-square = 20.09). MW2 versus USA400-0051 (*p *= 0.3186); MW2 versus 08–028 (p < 0.0001); MW2 versus 07–059 (*p *= 0.0043); USA400-0051 versus 07–059 (*p *= 0.1194); 08–028 versus 07–059 (*p *= 0.3186). For each strain, six experiments were set using five plates each, and 30–40 nematodes were added to each plate. Each data point is the mean of the data collected from these six experiments. The total number of worms considered in time zero (100%) was MW2 (n = 225); USA400-001 (n = 237); 07–059 (n = 181) and 08–028 (n = 264)

The archetypes of ST1-BR and ST1-USA strains were also compared in a *C. elegans* model. No important difference was found between US strains MW2 and USA400-0051 or between Brazilian strains 07–059 and 08–028. A comparison of MW2 and USA400-0051 (survival rate of 23.0% and 29.0%) and 08–028 and 07–059 (survival rates of 46.0% and 41.0%) showed more nematode death on day 3 (Figure 10).

## Discussion

Here, we show that although ST1 strains from Brazil and other countries conserve most of their virulence-associated genes, they do have differences in the presence/absence of well-characterized and putative virulence genes including *ear, sell, sec, selQ, selK, sea, fnbB*, and *lukSF-PV*. The CA-MRSA strains from the US carry all these genes while the vast majority of HA-MRSA from Rio de Janeiro carry none of them but do display a complete *spl* operon. A multitude of combinations for these genes was observed for the ST1 genomes analyzed. The gene content differences within the same ST or even more closely related lineages are not a new observation. Recently, for instance, we showed that ST239 strains from different geographic regions display specific and phylogeographic-pathotype patterns implying allopatric diversification and evolution [30].

Challangundla et al. [21) also found that CC5 HA-MRSA from the Western Hemisphere includes not only one but multiple prevalent clones that experienced convergent losses of the *sep* gene and specific losses of surface proteins. Also, important differences in virulence traits were found in USA300 CA-MRSA from North America (USA300) and Latin America (USA300-LV) [31]. However, the events of microevolution reported here are unique because they are associated with differences in ST1-SCC*mec*IV clinical manifestations: (i) infection of healthy individuals from the community; or (ii) hospital infections in immunocompromised patients. This provides potential new insights into how MRSA evolves to gain better fitness in each specific context.

The trees topologies clearly show that contrary to our prior hypothesis, there is no direct relationship between ST1 CA-MRSA from the US and ST1 HA-MRSA from Rio de Janeiro as previously suggested [7]. Most ST1-BR showed a gene signature that was more similar to European ST1 from Italy and Denmark grouped in the orange subclade except for the presence of *fnbB* gene in the Europeans. However, the tree architecture disclosed a close relation between these two clusters indicating a convergent (independent) evolution in the loss of these non-essential genes. Actually, the most frequently detected ST1-BR lineages are closely related to the MRSA strain LC33, which was isolated in 2012 from human milk in Bahia and has a gene signature identical to the vast majority of European ST1. It was estimated that LC33 and ST1-RJ shared a common ancestor in 1972 (95% HPD, 2003–1941). This might be an indication that the loss of *fnbB* was a relatively recent event coinciding with the dramatic spread of ST1-SCC*mec* IV in Rio de Janeiro hospitals.

Importantly, the phylogenetic tree clearly shows that not all ST1 carrying *lukSF-PV* belong to the MW2/USA400 clone. In addition, a more local dissemination seems to be more common among ST1 MRSA despite some intercontinental spread (e.g.: green subclade: ERS746482 from Thailand and BU_W6_t1 from Ghana; black: 211P from Italy and 1269 isolated from Brazilian cows; yellow: ST1509 from the UK and A1 from Australia; and red: ERS410852 from Thailand and 2288 from the US). The tree shape also suggests that many distinct acquisitions of SCC*mec*IV occurred in MSSA strains throughout the evolution of ST1, which is corroborated by the observation of different SCC*mec*IV types among ST1 genomes [31–33]. Similar patterns of dissemination were found for CC5 MRSA in which SCC*mec* elements appear to have been acquired in distinct events by MSSA strains with a subsequent spread in a more regional fashion [21]. Nevertheless, this pattern differed from ST239 MRSA for which SCC*mec*III seems to have entered only once or a few times with subsequent international spread followed by allopatric adaptation and local expansion [29].

This study highlights the combined importance of MGEs in the establishment of virulence (bacteriophages and genomic island SAPImw2) and susceptibility patterns (resistance-associated plasmid pMW2-like). In fact, the presence of a virulence gene in *S. aureus* is often associated with the acquisition of an associated mobile genetic element (MGE) [34]. However, it is striking that ST1-SCC*mec*IV, whether or not they were carrying ear, *lukSF-PV, sea, selK, selq*, or *splDE* always harbored the associated MGE in their genomes, which was often associated with a high nucleotide identity. This observation implies that the absence of virulence genes cannot be used as a direct indication of the absence of the correspondent MGE or vice-versa. Indeed, the analysis of the regions flanking the prophages PhiSa2MW and PhiSa3MW (200 nt upstream and downstream) carrying (or not carrying) the associated genes showed 100% nucleotide identity. This observation also suggests that the mechanism for the loss or gain of genes carried by phages is likely due to homologous recombination between sequences of highly similar bacteriophages – a process that has been proposed as operating in *S. aureus* phage mosaicism [35].

No ST1-BR from Rio de Janeiro carried the *fnbB* gene except CM 06/02, which has a virulence profile identical to the AU strain 0515798. It has been suggested that the N3 subdomain of the protein FnBPB enhances biofilm development, and the N2 domain promotes bacterial adhesion to host fibronectin [36]. Therefore, it is possible that the absence of FnBPB contributed to the reduction observed in biofilm accumulation and other colonization properties observed for representatives of ST1-BR.

Global downregulation of proapoptotic exoproteins had previously been associated with decreased virulence and with an increase in *S. aureus* persistence [37–39]. However, the contribution of Spl proteases for *S. aureus* virulence is not clearly understood. Therefore, we used MW2 cloned with *splD* or *splE* to investigate a possible advantage for the conservation of the s*plDE* genes by ST1-BR strains in the context of hospital infections. In agreement with the virulence attenuation observed for ST1-BR strains [7, and this work], our data suggest that *splD* plays a role in reducing cytotoxicity with a parallel increase in intracellular survival, similarly to that observed for the archetypal strain 08–028 (ST1-BR) when compared with strain MW2 [ST1-US). The importance of *splD* in the *S. aureus* pathogenicity is not well defined. Studies based on the SplD cleavage site consensus variant found 100 hits in the proteome of*Homo sapiens* including the tumor necrosis factor ligand superfamily member 13B (Q9Y275], which can direct or indirectly regulate the differential expression of a variety of genes involved in the innate response and the regulation of apoptosis as well as a number of other cytoplasmic and membrane proteins [40]. In addition, they found four hits in *S. aureus*: antibiotic epidermin biosynthesis protein EpiB (Q2FXB3), putative ferrous iron transport protein B (Q2FV72), putative uncharacterized protein SAOUHSC_00304 (Q2G151), and putative uncharacterized protein SAOUHSC_01866 (Q2FXI1). Consequently, one may suppose that SplD could be degrading either a *S. aureus* proapoptotic factor or a host protein important in the apoptosis pathway. Nevertheless, the mechanism by which the *splD* gene affects bacterial cytotoxicity and cell persistence in the ST1 background remains to be clarified.

In fact, based on the scientific literature [37–39], it is reasonable to infer that the absence of genes encoding several enterotoxins, enterotoxin-likes, and the Panton-Valentine leucocidin in the genome of the vast majority of ST1-BR may have also reduced the cytotoxicity with an increase in the intracellular survival observed in this work. Indeed, the high tendency of low cytotoxic isolates to cause bacteremia has been previously demonstrated [39,41]. This phenomenon seems paradoxical due to the role of these toxins in *S. aureus* pathogenesis. For example, using a *sec* knockout derived from the CA-MRSA MW2 [42], it was demonstrated that the encoded enterotoxin, SEC, is important in the development of sepsis, endocarditis, and renal damage in animal models. In addition, the presence of SPImw2 carrying *sec* was associated with skin soft tissue infections [43]. Other studies, using *sea* knockouts, indicated that this toxin exacerbates staphylococcal infections by inducing renal abscess formation in a bacteremia model in mice; the toxin also increases the influx of pulmonary neutrophils [44]. SEA can also modulate myeloid-derived suppressor cells (MDSC) in a dose-dependent manner with high concentrations showing profound cytotoxic activity whereas low concentrations had a proliferative effect on MDSCs [45]. SELK also plays a role in abscess formation and consequently contributes to the pathogenesis of skin and soft tissue infections, which are the most common manifestations of CA-MRSA strains [43].

In fact, the correlation between cytotoxicity and intracellular survival in *S. aureus* has been well documented [37–39,46]. Some studies have already associated these properties with an improved ability of *S. aureus* to cause disseminated infections such as bloodstream infections (BSIs) [37,38]. Mutations in the global virulence regulators *sarA* and *agr* increase *S. aureus* intracellular survival and decrease bacterial cytotoxicity [37,39]. Both *sarA* and *agr* downregulate several exoproteins with recognized proapoptotic activity including various enterotoxins, enterotoxin-like proteins, and PVL [37,47]. Likewise, persistent BSIs caused by MRSA are frequently attributed to *agr* attenuation, which may impact clinical outcomes [47,48]. Notably, *S. aureus* mutants in *rsp* – repressor of a surface protein and another global regulator that upregulates exoproteins – were mainly associated with patients that switched from nasal carries to BSIs [37].

Remarkably, *rsp* mutants showed attenuated cytotoxicity with increased intracellular survival inside host cells including neutrophils and reduced disease severity at the beginning of the experimental infections in animal model without compromising the bacterial ability to cause internal abscesses and BSIs [37]. It is interesting that *rsp* mutants in *S. aureus* were also detected in an unbiased genome-wide approach designed to identify genes mediating prolonged intracellular survival possibly due to attenuated cytotoxicity observed in parallel [37].

It is likely that the multiple drug resistance traits in ST1-BR also represent an additional adaptive advantage for the spread of ST1-BR in the hospital environment [7]. Multidrug resistance is common among MRSA involved in hospital infections though more susceptible CA-MRSA strains can also share the hospital environment and coexist with the more resistant HA-MRSA [5,49]. A loss of virulence genes may represent a trade-off in fitness in the context of multiple resistance. In fact, previous studies have demonstrated that the inactivation of virulence-associated genes may lead to an overgrowth of *S. aureus in vivo* in the abscess environment. Notably, these authors highlighted *sarA*, a positive regulator of enterotoxin, among the mutated genes examined [50].

## Conclusions

Our results suggest that the inability to cause community-acquired infections observed during the expansion of ST1 strains in Rio de Janeiro correlates to a reductive evolution of the virulence repertoire. It is possible that this strategy provides bacteria an advantage of increasing persistence in human host, especially considering bacterial transmission and infections among already debilitated patients, such as those who are hospitalized. Conversely, ST1 CA-MRSA from North America evolved to conserve their virulence and toxic capability which is compatible with the bacterial ability to cause acute infections in healthy individuals. Our data collectively point to strong and opposing selective regimes acting in the community and hospital settings as well as evolutionary “agility” that enables strains of *S. aureus* to adapt to different niches. Genomic studies aiming at understanding the evolution of *S. aureus* pathogenicity and the dynamics of the clonal diversity and spread are of utmost importance to unveil key mechanisms that may contribute to the fitness of emerging and successful MRSA clones. Further studies are guaranteed to elucidate the precise role of the serine proteases SplD and SplE in bacteria-host interactions and their importance in the context of community and hospital infections.

## Supplementary Material

Supplemental Material

## Abbreviations

BFS: bovine fetal serum; BHI: brain heart infusion; BRIG: blast ring image generator; BSIs: bloodstream infections; BU: biofilm unit; CA-MRSA: community-acquired methicillin-resistant *Staphylococcus aureus*; CDC: Centers for Diseases Control and Prevention; CFU: colony-forming unit; HA-MRSA: hospital-acquired methicillin-resistant *Staphylococcus aureus*; HPD: high superior density intervals; IS: insertion sequence; LDH: lactate dehydrogenase; MCMC: Markov Chain Monte Carlo; MGE: mobile genetic element; ML: maximum likelihood; MOI: multiplicity of infection; MRSA: methicillin-resistant *Staphylococcus aureus*; NA: nalidixic acid; NGM: nematode growth medium; PFGE: pulsed-field gel electrophoresis; PI: propidium iodide; PVL: Panton-Valentine leucocidin; RGP: region of genomic plasticity; SCC*mec*: staphylococcal cassette chromosome *mec*; SNPs: single nucleotide polymorphisms; ST: sequence typing; ST1c: completely closed genomes of ST1 strains; TE: transposase element; TSB: tryptic soy broth; WA-1: Western Australia-1 clone.
