# Supplementary material for: Reductive evolution of virulence repertoire to drive the divergence between community- and hospital-associated methicillin-resistant Staphylococcus aureus of the ST1 lineage
Source: Virulence. 2021 Mar 18;12(1):951–67. doi: 10.1080/21505594.2021.1899616 (PMC7993186; doi:10.1080/21505594.2021.1899616)
Supplement: Supplemental Material [file KVIR_A_1899616_SM3087.pdf]

Supplementary Table S1. Clinical data and *spa* typing of the MRSA strains belonging to ST1-SCC*mec*IV lineage sequenced in this study as well as the ST1 strain MW2 that has a completely closed genome and was deposited in the GenBank.

Strain	Isolation year	Spa *type*	Clinical site	MRSA type	Country
**MW2 ●**	1998	t128	Blood	CA-MRSA[b]	USA
**USA400-0051●**	2003-2006	t128	Skin/Soft tissue	CA-MRSA	USA
**2288 ●**	NR[a]	t125	NR	CA-MRSA	USA
**08-028 ●**	2005-2009	t127	Blood	HA-MRSA[c]	Brazil
**07-059 ●**	2005-2009	t127	Blood	HA-MRSA	Brazil
**950122 ●**	2009-2010	t128	Blood	CA-MRSA	Canada
**111250134 ●**	2011-2012	t128	Blood	CA-MRSA	Canada
**515798 ●**	28/03/2005	t128	Nose	CA-MRSA	Australia
**CR 14-005 ○**	15/07/2014	t127	Nose	HA-MRSA	Brazil
**CR 14-006 ○**	15/07/2014	t127	Nose	HA-MRSA	Brazil
**CR 14-039 ○**	21/09/2014	t127	Nose	HA-MRSA	Brazil
**CR 14-040 ○**	28/09/2014	t127	Blood	HA-MRSA	Brazil
**CHU 15-072 ○**	29/05/2015	t127	Blood	HA-MRSA	Brazil
**CHU 15-073 ○**	12/06/2015	t127	Blood	HA-MRSA	Brazil
**CHU 15-090 ○**	30/06/2015	t127	Nose	HA-MRSA	Brazil

[a]NR: Not reported;
[b]CA-MRSA: Community-acquired MRSA;
[c]HA-MRSA: Hospital-acquired MRSA;
●: completely closed genome; ○: assembled genome.

Supplementary Table S2. Clinical data of the 53 Brazilian ST1-SCC*mec*IV used for the DNA-Microarrays analysis.

Isolate	Infection site	Isolation year
CM 06/02	Blood	2006
CM 05/35	NA[a]	2005
CM 05/65	Blood	2005
CM 05/69	Blood	2005
CM 05/89	Blood	2005
CM 05/96	Catheter tip	2005
CM 05/97	Blood	2005
CM 05/101	Nose	2005
CM 05/105	Nose	2005
CM 05/106	Nose	2005
CM 05/117	Blood	2005
CM 06/50	Blood	2006
CM 06/58	Skin and soft tissues	2006
07-029	Blood	2007
07-030	Blood	2007
07-033	Nose	2007
07-035	Nose	2007
07-042	Blood	2007
07-057	Catheter tip	2007
07-062	Nose	2007
07-121	Bronchial aspirate	2007
07-123	Bronchial aspirate	2007
07-135	Blood	2007
07-138	Blood	2007
07-153	NA	2007
07-158	Blood	2007
07-159	Nose	2007
07-160	Blood	2007
07-163	Blood	2007

08-001	Blood	2008
08-008	Blood	2008
08-010	Blood	2008
08-012	Bronchial aspirate	2008
08-014	Catheter tip	2008
08-17	NA	2008
08-018	Bronchial aspirate	2008
08-027	Catheter tip	2008
08-031	Skin and soft tissues	2008
08-036	Blood	2008
08-039	Catheter tip	2008
08-041	Catheter tip	2008
08-043	Blood	2008
08-051	Urine	2008
08-052	Blood	2008
08-054	Catheter tip	2008
08-059	NA	2008
08-063	Blood	2008
08-065	Blood	2008
08-066	Blood	2008
08-067	Blood	2008
08-073	Blood	2008
09-101	NA	2009
09-105	Catheter tip	2009

[a]NA: Data not available.

Supplementary Table S3. Clinical data of the 81 ST1-INT genomes deposited in the GenBank used for tree constructions and other analysis; there were four ST80 genomes (SA5-LAU, SA6-LAU, SA7-LAU, and SA12-LAU) used for rooting the LM tree.

Isolate	Acession	Isolation year	Isolation source	Region
**MRSA ERS410852**	SAMEA2384527	2009	disease	USA
**MSSA ERS410581**	SAMEA2384256	2009	disease	USA
**st2786**	SAMEA1464343	2010	blood	United Kingdom: England
**st2099**	SAMEA1317313	2008	blood	United Kingdom: England
**st1881**	SAMEA1317392	2008	blood	United Kingdom: England
**st1509**	SAMEA1464324	2007	blood	United Kingdom: Northern Ireland
**st1070**	SAMEA1464707	2005	Blood	United Kingdom: England
**st304**	SAMEA1464549	2002	blood	United Kingdom: England
**st1561**	SAMEA1464588	2002	blood	United Kingdom: England
**st1315**	SAMEA1469688	2006	blood	United Kingdom: England
**st314**	SAMEA1464506	2002	blood	United Kingdom: England
**1269**	LNOO00000000	2014	Milk	Brazil
**BU_W6_t1**	NZ_LFNO00000000	2012	wound	Ghana: Pakro
**A1**	SAMN03753597	2004	lungs of cystic fibrosis	Australia: Brisbane
**MSSA476**	NC_002953.3	1998	osteomyelitis and bacteremia	United Kingdom
**169_SAUR**	NZ_JVRM00000000.1	NA[a]	NA	USA:WA
**327**	SAMN02983094	2013	right forehead swab	Malaysia
**337**	GCA_000763455.1	2013	ear swab	Malaysia
**CO-41**	NZ_JHQE00000000.1	NA	Na	NA
**21304**	SAMN00117451	NA	NA	NA
**S2396**	NZ_CAWA000000000	2005	NA	France:Lyon
**MUM475**	AZSG00000000.1	2012	wound fluid	India
**S2395**	NZ_CAVU000000000.1	2005	NA	France:Lyon
**MUF256**	AZSE00000000.1	2012	wound fluid	India
**KT/314250**	NZ_AOCP00000000.1	2008	pus	Malaysia: Terengganu
**CIG1835**	AIEQ00000000.1	/2012	NA	NA
**CIGC128**	AHVY00000000.1	2003	nasal swab	USA: North Carolina, Durham
**TCH70**	NZ_ACHH00000000.2	NA	NA	texas
**ATCC51811**	SAMN00139434	2010	NA	NA
**GKP138-41**	NZ_FMQC00000000.1	NA	NA	NA
**MRSA012TA**	NZ_FMPB00000000.1	NA	NA	NA
**MRSA066**	NZ_FMOL00000000.1	NA	NA	NA
**MSSAT94**	NZ_FMSF00000000.1	NA	NA	NA
**Sa12-002**	NZ_MAQH00000000.1	2012	milk filter	Australia: Victoria
**Sa14-001**	NZ_MAQO00000000.1	2014	milk filter	Australia: Victoria
**ERS746560**	SAMEA3448998	2015	NA	Thailand
**ERS746512**	SAMEA3448950	2015	NA	Thailand
**ERS746438**	SAMEA3449400	2015	NA	Thailand
**ERS746399**	GCA_900125475.1	2015	NA	Thailand
**ERS746482**	GCA_900126045.1	2015	NA	Thailand

**ERS746630**	GCA_900126535.1	2015	NA	Thailand
**ERS746389**	GCA_900127805.1	2015	NA	Thailand
**ERS746522**	GCA_900128095.1	2015	NA	Thailand
**CM135**	GCA_900126045.1	2015	sputum	Italy: Florence
**CM127**	GCA_003237355.1	2015	bronchoaspiration material	Italy: Florence
**CM109**	GCA_003237515.1	2013	bronchoaspiration material	Italy: Florence
**CM124**	GCA_003238125.1	2015	bronchoaspiration material	Italy: Florence
**CM91**	GCA_003238315.1	2014	various material	Italy: Florence
**CM81**	GCA_003238375.1	2013	bronchoaspiration material	Italy: Florence
**CM64**	GCA_003238485.1	2015	pharyngeal swab	Italy: Florence
**CM29**	GCA_003239715.1	2014	sputum	Italy: Florence
**CM53**	GCA_003238595.1	2015	pharyngeal swab	Italy: Florence
**CM26**	GCA_003238805.1	2014	sputum	Italy: Florence
**H1446**	GCA_900250415.1	NA	NA	Denmark: Copenhagen
**HL452**	GCA_900250795.1	NA	NA	Denmark: Copenhagen
**M3117**	GCA_900251105.1	NA	NA	Denmark: Copenhagen
**M3998**	GCA_900252035.1	NA	NA	Denmark: Copenhagen
**RH259**	GCA_900252205.1	NA	NA	Denmark: Copenhagen
**549**	GCA_002798125.1	2016	Blood stream	Brazil: Parana
**UA825**	GCA_002887375.1	2013	blood	Argentina: Buenos Aires
**ch24**	GCA_003336555.1	2008	broiler chicken	Poland: Wroclaw
**LC33**	GCA_001921665.1	2012	milk sample	Brazil: Bahia
**211P**	GCA_002572965.1	2016	raw milk	Italy: Bari
**FORC_026**	GCA_001879545.1	2014	blood	South Korea: Seoul
**FORC_045**	GCA_002209325.1	2014	blood	South Korea: Seoul
**CFSAN007851**	GCA_002633825.1	2011	bakery environment	USA: IL
**JMUB3031**	GCA_003573835.1	2017	subcutaneous abscess	Japan
**SCPM-O-B-7907**	GCA_002188415.1	2015	NA	NA
**SCPM-O-B-7904**	GCA_002188495.1	2015	NA	NA
**698**	GCA_002096985.1	2009	Carriage sample	Australia
**B26B_160215**	GCA_002905535.1	2015	blood	Ghana: Korle Bu
**SAM-16**	GCA_003038455.1	2016	wound	Lebanon
**SAM-9**	GCA_003038575.1	2016	tracheal aspiration	Lebanon
**SAM-8**	GCA_003038595.1	2016	tracheal aspiration	Lebanon
**3688STDY6124879**	GCA_900126415.1	2015	NA	Thailand
**CFSA074**	GCA_002125325.1	2014	sputum or oropharyngeal	USA: Cincinatti
**0908-2002-2015**	GCA_003017755.1	2015	Skin lesions	Russia: Moscow
**MSSA_FKXU011**	GCA_900083415.1	2010	wound	USA
**ST20110167**	GCA_900149335.1	NA	blood	NA
**H1688**	GCA_900250665.1	NA	NA	Denmark: Copenhagen
**M2885**	GCA_900250985.1	NA	NA	Denmark: Copenhagen
**SA6- LAU**	JHDX00000000.1	2007	Wound	Jordan: Amman
**SA5-LAU**	NZ_JHDW00000000.1	2007	Wound	Jordan: Amman

**SA12-LAU**	NZ_JHED00000000.1	2007	Wound	Lebanon: Byblos
**SA7-LAU**	NZ_JHDY00000000.1	2011	Abscess	Lebanon: Byblos

[a] NA: Data not available.

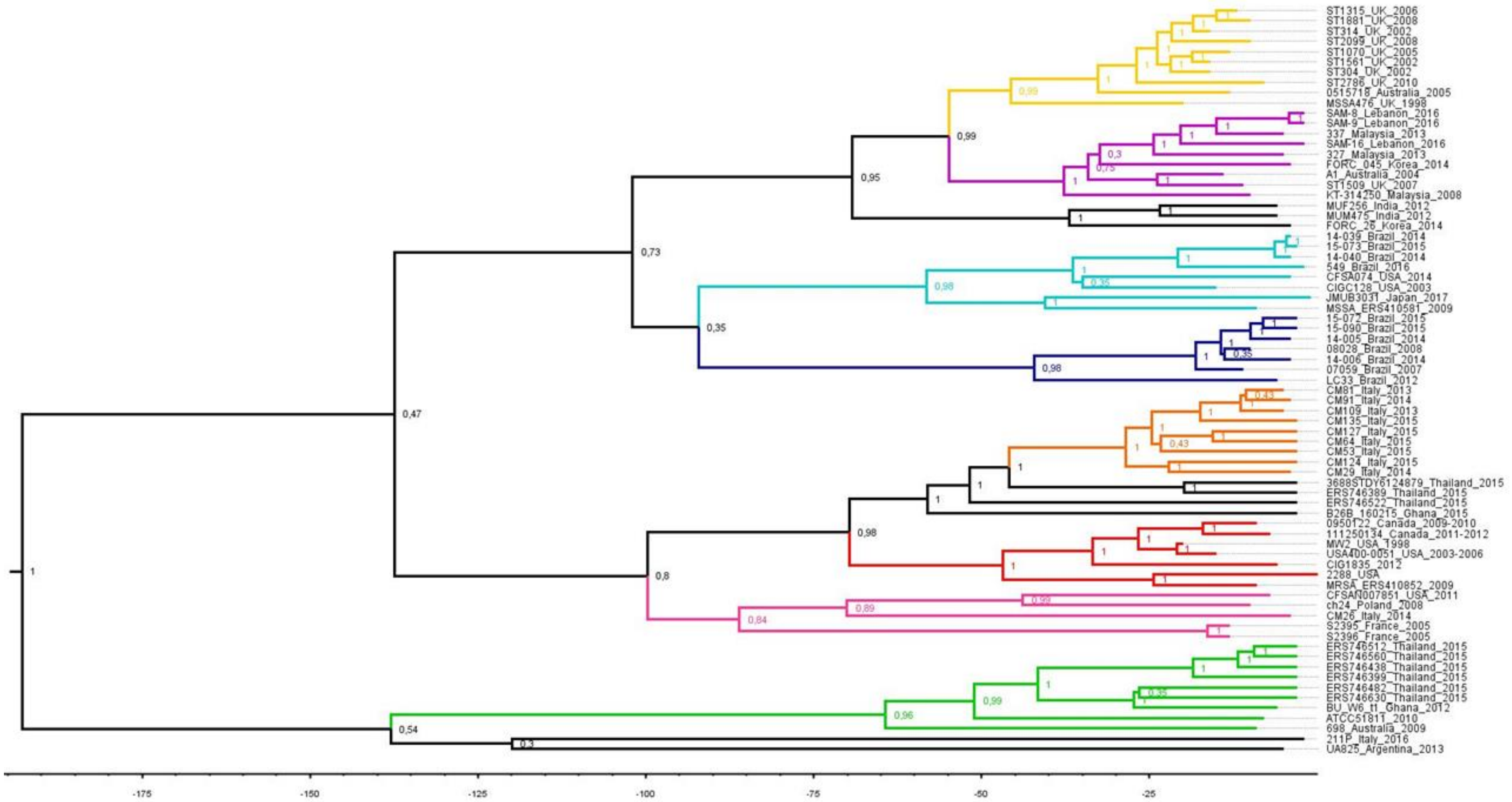

Supplementary Figure S1. The evolutionary history of ST1 strains of MRSA was based on a Bayesian inference assuming a relaxed clock and general time reversible nucleotide substitution. The main subclades were highlighted with different colors in line with the Figure 4. Posterior probability support for each node is indicated in the tree. The branches are scaled in years (indicated under the tree).

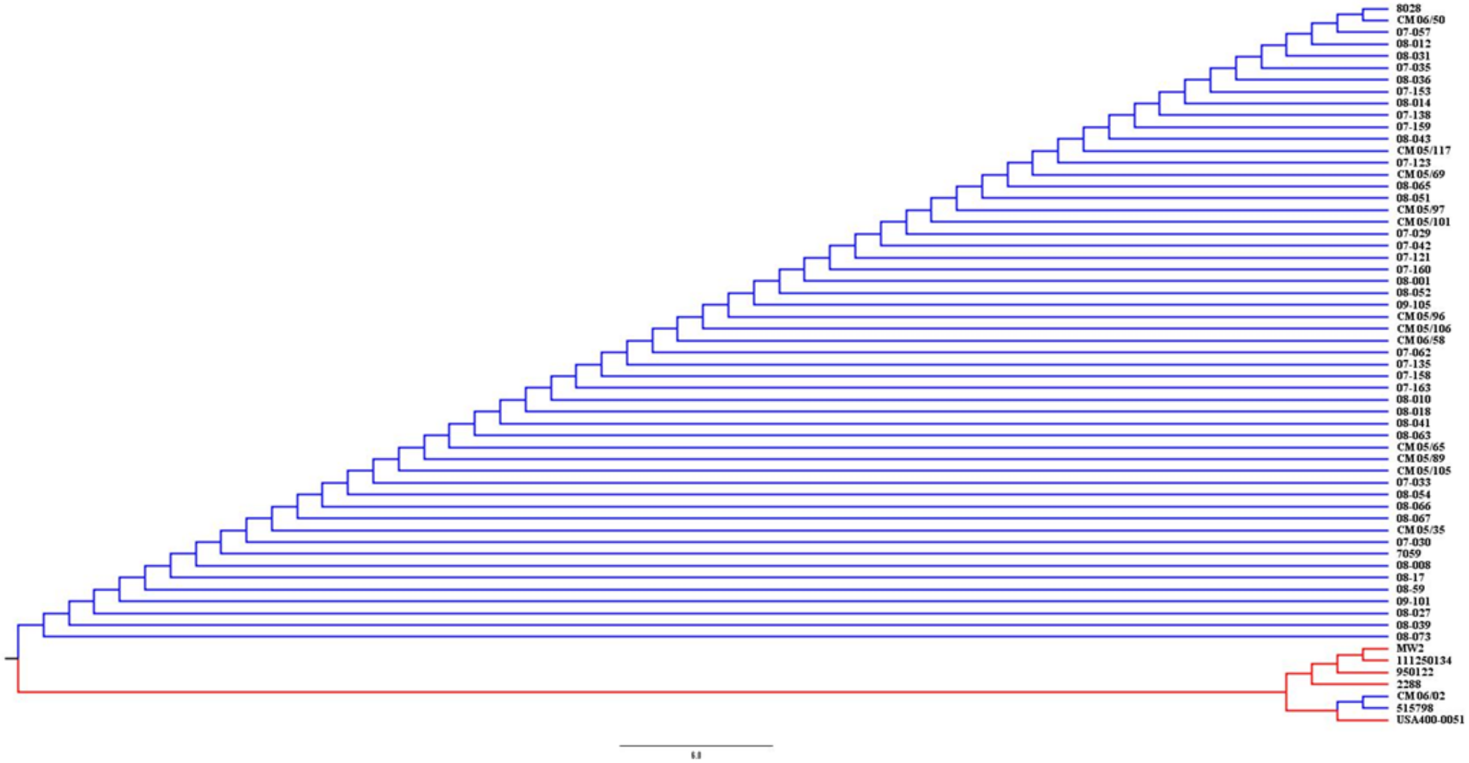

Supplementary Figure S2. Hierarchical binary cluster tree based on DNA-microarray data. Strains MW2, USA400-0051, and 2288 (EUA), strains 111250134 and 950122 (CA), strain 0515798 (AU), and all other strains listed are from Brazil.

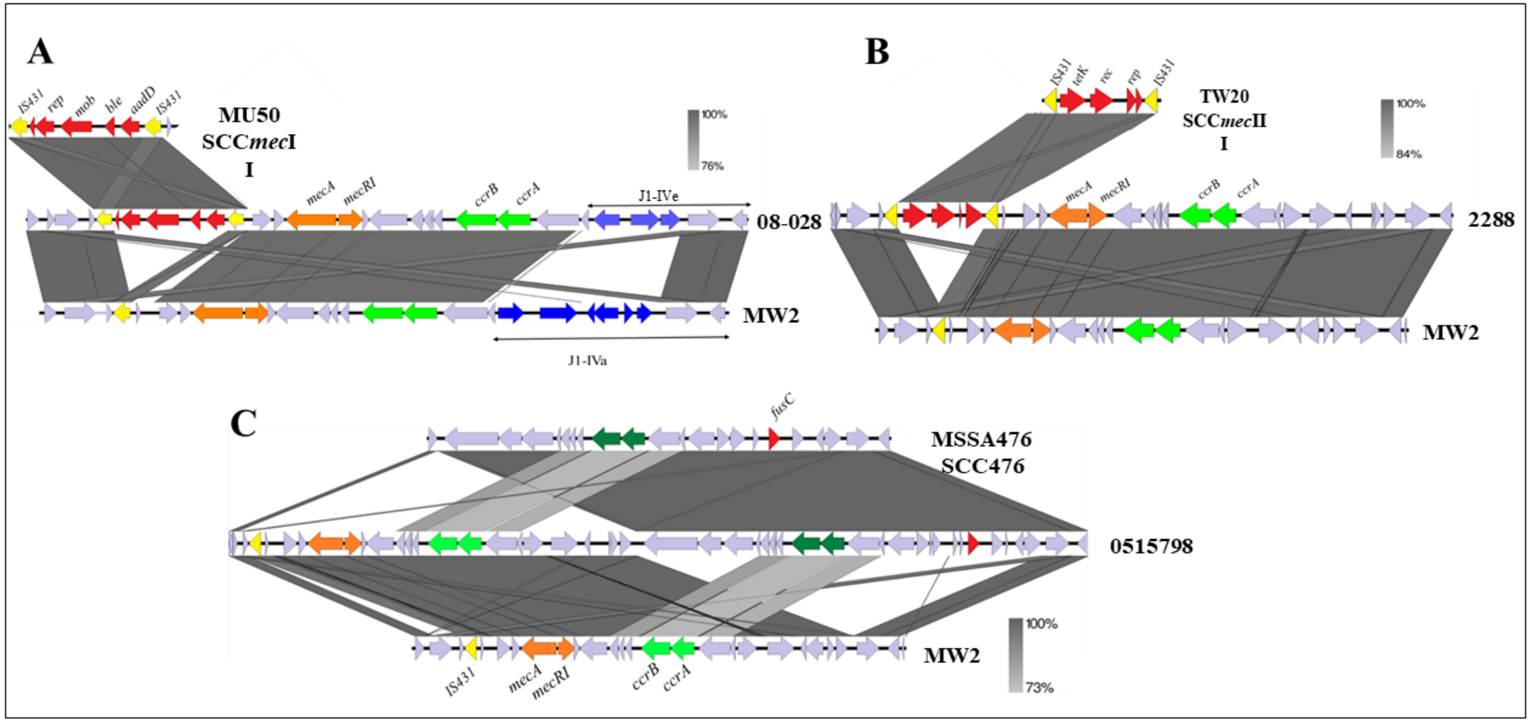

Supplementary Figure S3. Genetic context of different SCC*mec*IV regions in ST1 genomes suggesting SCC*mec* recombination. **A**. Comparison of SCC*mec*IV from 08-028 (ST1-BR) with that of MW2 (ST1-USA) and with part of the SCC*mec*II of the strain MU50 (ST5 from the USA whose genome is available in the GenBank). **B**. Comparison of the SCC*mec*IV of the strain 2288 with that of MW2 and with part of the SCC*mec*III from the strain TW20 (ST239 from the UK whose genome is available in the GenBank). **C**. SCC*mec*IV of the ST1 strain 0515798 (ST1-AU) with that of MW2 and with part the SCC476 from the strain MSSA476 whose genome is available in the GenBank.

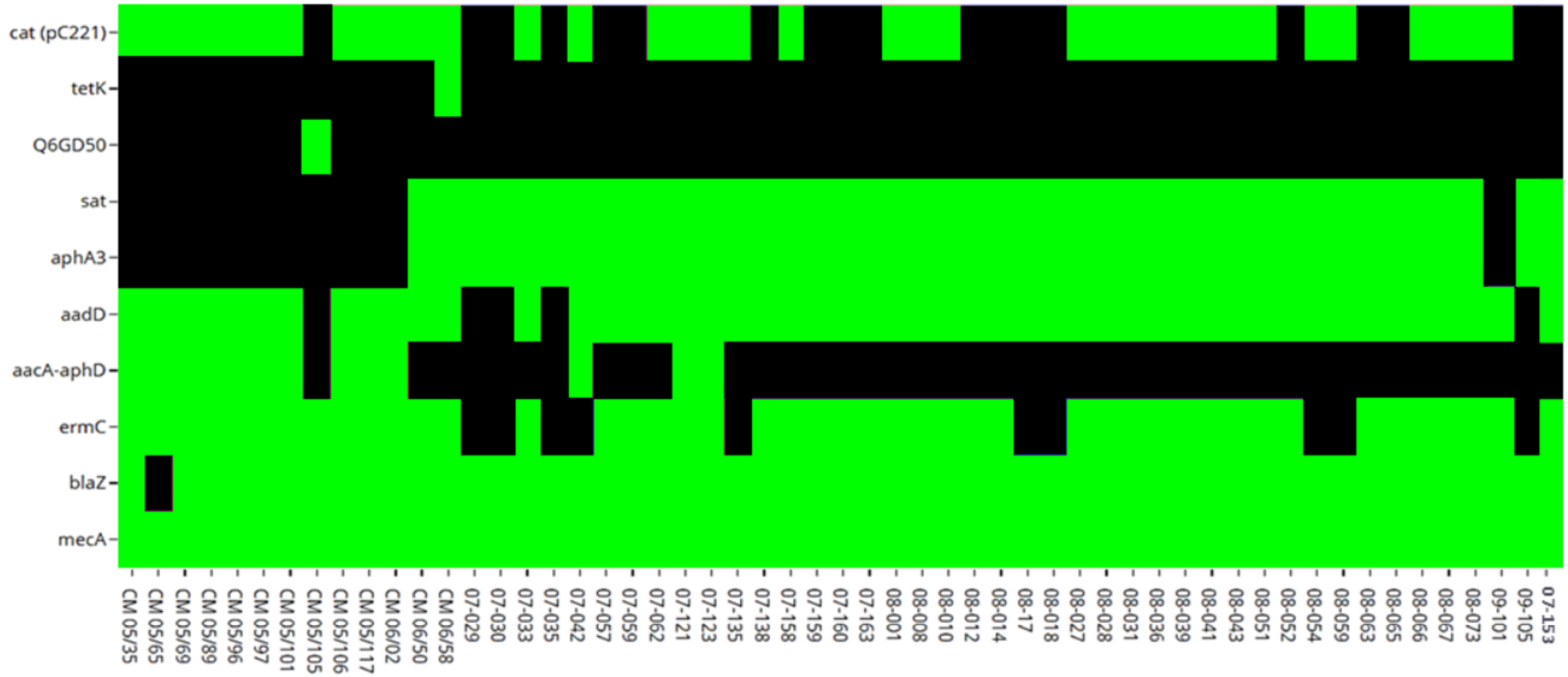

Supplementary Figure S4. Heatmap of the resistance-associated genes of ST1-BR strains detected by DNA-microarrays. **Green**: gene presence, **black**: gene absence. ***cat*** (pC221): chloramphenicol resistance in pC221 plasmid; ***tetk***: tetracycline resistance; q***6g50***: hypothetical protein associated with fusidic acid resistance; ***sat***: streptothricine resistance; ***aphA3***: aminoglycoside phosphotransferase/kanamycin resistance; ***aadD***: aminoglycoside adenyltransferase/tobramycin resistance; ***aacA-aphD***: bifunctional enzyme Aac/Aph/gentamicin resistance; ***ermC***: erythromycin/clindamycin resistance; ***blaZ***: β-lactamase/ penicillin resistance; and ***mecA***: methicillin resistance.
